# A Comparative Study of Single-Layered Versus Double-Layered Intestinal Anastomosis

**DOI:** 10.7759/cureus.23088

**Published:** 2022-03-12

**Authors:** Rommel Singh, Husain I Najmi, Reetinder K Chahal, Dehankar Nikhil

**Affiliations:** 1 Department of General Surgery, Government Medical College (GMC) and Rajindra Hospital, Patiala, IND

**Keywords:** postoperative intestinal obstruction, postoperative complications, single-layered, double-layered, anastomosis

## Abstract

Background: Intestinal perforations requiring resection anastomosis of the gastrointestinal tract (GIT) or the formation and closure of temporary intestinal stoma are prevalent worldwide. This prospective comparative study was done to assess the efficacy and safety of single-layered anastomosis compared to a double-layered anastomosis.

Methods: Patients undergoing intestinal anastomosis with either of these two techniques were observed prospectively for various outcome parameters like time taken for anastomosis, and that for entire surgery, postoperative complications, etc. Data obtained were analyzed for statistical significance by applying the chi-square test and student's “t-test.”

Results: Duration for fashioning the anastomosis was significantly lesser for a single layer anastomosis than double (mean [±SD] for single layer was 19.57 ± 2.25 minutes and for double layer group was 30 ± 2.59 minutes, p=0.002). There was no statistical difference in the postoperative complications between the two groups. The postoperative incomplete intestinal obstruction was reported in three cases of the double layer group.

Conclusion: Single-layered gastrointestinal anastomosis (GIA) resulted in a significant reduction in time, without any difference in complications. Additionally, it is easier to train surgical residents in the single-layered technique which is particularly important in the setting of a teaching institute and can be recommended for intestinal anastomosis.

## Introduction

Intestinal perforations due to infectious diseases, trauma, malignancy, etc., requiring resection-anastomosis of the gastrointestinal tract (GIT) or formation and closure of a temporary intestinal stoma are prevalent procedures in India. Currently, expert management of intestinal perforation is based on a detailed knowledge of epithelialization, protein synthesis, the biology of wound contraction, the surrounding milieu of the healing wound, ground substance, and the factors which affect healing [1,2]. The reported rate of failure of gastrointestinal anastomosis (GIA) ranges from 1.5% to 2.2%, depending on what type of anastomosis was performed, where the operation was elective or an emergency procedure, general factors as age, nutritional status, comorbid conditions, and local factors like vascularity, sepsis, and suture technique. A leaking anastomosis dramatically increases the morbidity and mortality associated with the operation: it can increase the length of hospital stay, the total cost of the treatment, and mortality [3].

It has been a debatable issue regarding the outcomes of single-layered or double-layered anastomosis. In many conditions, double-layered anastomosis consists of a continuous transmural inner layer and outer inverted seromuscular layer. However, double-layered anastomosis carries the disadvantage of mucosal inversion and serosal apposition. The inner layer is believed to be hemostatic but due to damage to the submucosal vascular plexus, it leads to mucosal strangulation. Currently, single-layer intestinal anastomosis is popular as it probably causes less tissue necrosis or luminal narrowing and requires less time and cost without adding the risk of leakage [4,5].

The innovations mentioned above in patient care and safety enabled a series of development to follow in improving the outcomes of elective and emergency intestinal anastomosis, leading up to today’s current standards of practice.

## Materials and methods

The study was conducted after permission from the institutional ethics committee with vide letter no. “(TRG)EC/NEW/.INST/2020/997/4417.” In this comparative study, 60 patients in which intestinal anastomosis was done in the Department of General Surgery, Government Medical College (GMC) and Rajindra Hospital Patiala were prospectively randomized by chit and box method into two groups of 30 each. In one group, single layer intestinal anastomosis (group A) whereas in the other group, double-layered (group B) intestinal anastomosis was done.

The study assessed the efficacy of both groups in terms of the duration required to perform single and double-layered intestinal anastomosis, study post-operative complications like an anastomotic leak (AL) in the single and double-layered intestinal anastomosis, the outcome associated with single and double-layered anastomoses and the duration of hospital stay in either of them and safety of single-layered anastomosis compared to a double-layer anastomosis.

The technique of single-layered intestinal anastomosis comprised the single-layered intestinal anastomoses performed using a 3-0 polyglycolic acid suture by interrupted extra mucosal approximation of the cut end of the intestine while the technique of double-layer intestinal anastomosis leads to a transmural continuous suture layer applied with absorbable 3-0 suture material like polyglycolic acid thereafter followed by an interrupted, seromuscular suture layer with a non-absorbable 3-0 silk suture.

Inclusion criteria of the study were all patients admitted to the Surgery ward in GMC and Rajindra Hospital, Patiala, in which intestinal anastomosis was required which are hemodynamically stable and who had given informed consent. The patients with co-morbid conditions like diabetes mellitus (DM), hypertension (HTN), immunodeficiency and bleeding disorders, patients who underwent stapled anastomosis, patients with sepsis (defined as a sequential organ failure assessment [SOFA] score >/= 2 with a known focus of infection), septic shock (sepsis with a need for vasopressors to maintain MAP >/= 65 mmHg), cachectic patients (loss of >/= 5% of body weight in the last 12 months or less, with a known illness) on admission or those requiring total parenteral nutrition (TPN) and intensive care unit (ICU), patients who developed any complications due to anesthesia and other pre-existing renal, pulmonary and cardiac complications were excluded from this study.

## Results

The overall mean ±SD age was 36.6 ±15.37 years, and that of the patients in groups A and B was 38.6 ±15.85 and 34.63 ± 14.87 years, respectively. Males predominated our study population. Forty-three patients (71.7%) were males, of whom 21 (35%) were from group A, and 22 (36.7%) were from group B. Seventeen females were included in the study (28.3%), of whom nine (15%) belonged to group A and eight belonged to group B (13.3%). The patients’ mean (±SD) weights from groups A and B were 64.16 ± 10.14 and 60.73 ± 8.23 kg, respectively. The mean hemoglobin (Hb) of the patients in our study was 11.54 ± 1.19 g/dL. Sixty percent of patients (36 patients) had Hb ranging from 10 to 12 g/dL. Only four patients (7%) had Hb below 10 g/dL (Table 1).

**Table 1:** Variables of the present study *SL = Single-layered; **DL = Double-layered

Variables	Grp. A (*SL)	Grp. B (**DL)	Total	P-value
Mean Age Distribution (in years)	38.6	34.63	36.61	0.322
Gender Distribution (M=Male; F=Female)	M=21/30, F= 9/30	M=22/30, F= 8/30	M=43/60, F= 17/60	0.774
Bodyweight (in kilograms)	64.16±10.14	60.73±8.23	62.44±9.32	0.156
Mean Hemoglobin (in g/dL)	11.46±1.44	11.6±0.90	11.54±1.19	0.577
Total Serum Proteins (in gm/dL)	6.10±0.71	6.62±0.50	6.35±0.66	0.002
S. Albumin (in g/dL)	3.19±0.32	3.15±0.30	3.16±0.30	0.636
Type of anastomosis				0.363
Colo – colic anastomosis (Number)	1	1	2	
Colo – rectal anastomosis (Number)	2	2	4	
Entero – colic anastomosis (Number)	4	1	5	
Entero – enteric anastomosis (Number)	23	26	49	
Duration of anastomosis (in minutes)	19.57±2.25	30±2.59	24.78±5.78	0.001
Complications				
Abscess (Number)	2	2	4	0.888
Fistula (Number)	1	1	2	
Intestinal Obstruction (Number)	0	3	4	
Leak (Number)	2	2	4	
Wound Infection (Number)	4	7	11	
Wound Dehiscence (Number)	3	3	6	
Duration of hospital stay (in days)	8.97±3.08	8.93±2.61	8.95±2.83	0.964
Follow up	Non-specific =28	Non-specific =28	Non-specific =56	0.513
	Recovered=2	Recovered=1	Recovered=3	
	Death=0	Death=1	Death=1	

The mean total serum protein (TSP) of the patients in our study was 6.35 ± 0.66 g/dL. Thirty-three patients had a TSP from 7 to 7.9 g/dL (55%). Only two patients (7%) from group A had TSP < 5 g/dL. On statistical analysis, the difference in the TSP between the two groups was statistically significant (p=0.002). The mean serum Albumin (S. Alb) concentration of the patients in our study was 3.16 ± 0.30 g/dL. The difference in the S. Alb concentration between the two groups was found to be statistically insignificant (Table 1).

**Figure 1:**
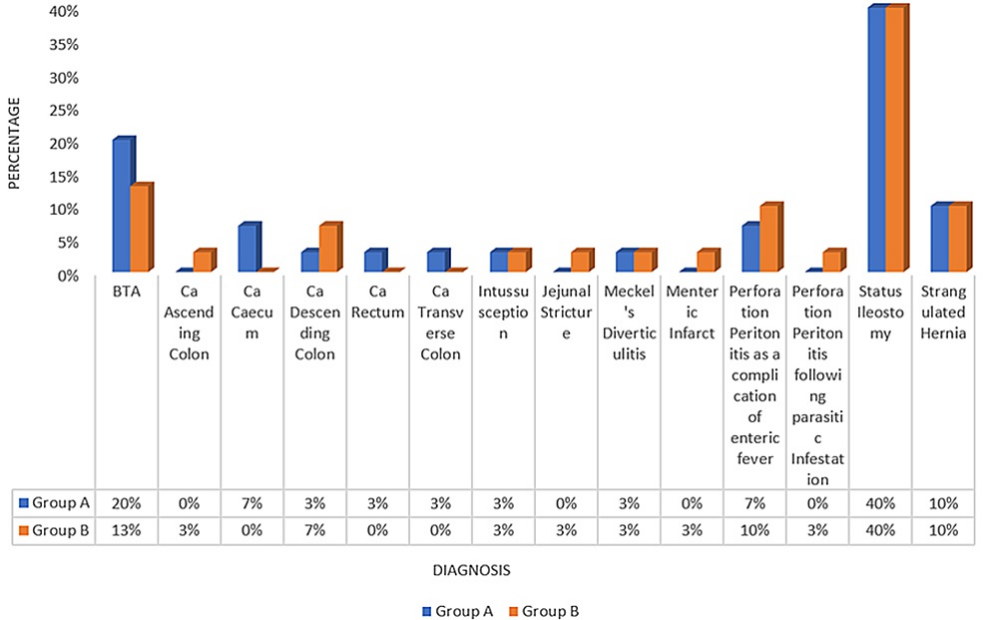
Diagnosis of patients BTA = Blunt trauma abdomen; Ca = Carcinoma

The most common indication for anastomosis was status ileostomy, in 24 patients (40%), of whom 12 patients were from both groups A and B (40%). Consequently, the most common procedure done in our study was ileostomy closure, done in 24 patients (40%). The commonest type of anastomosis was enteroenteric anastomosis in 49 patients (81.6%), with 23 patients in group A (76%) and 26 patients in group B (87%). It was followed by enterocolitis anastomosis in five patients (8%), with four of them being in group A (13%) and one being in group B (3%). Colorectal anastomosis was done in four patients (7%), and colocolic anastomosis was done in two patients (3%), respectively (Figure 1). The mean duration of anastomosis for group A was 30 ± 2.59 minutes and for group B was 19.57 ± 2.25 minutes. On statistical analysis, the duration of anastomosis was highly significant (p=0.002) (Figure 1).

The commonest complication encountered in the series was wound infection in four patients in group A (13%) and seven patients in group B (23%). It was followed by wound dehiscence in three patients in group A (10%) and three in group B (10%). Two patients each in groups A and B (7% each) developed an intra-abdominal abscess. In comparison, one patient each in groups A and B developed an enterocutaneous fistula (3%). Two patients each in groups A and B developed a leak (7%), for which they underwent re-exploration. On statistical analysis, the complications were insignificant (p=0.541) (Figure 1). Three patients in group B developed post-operative partial intestinal obstruction, which was managed conservatively (Figure 1).

On analysis, the difference in the incidence of postoperative complications was not statistically significant. One patient with post-operative leakage in group B expired during the hospital stay (1.6%). Three patients (5%) who were re-explored (two from group A and one from group B) recovered uneventfully and had an insignificant follow-up visit (Figure 1).

## Discussion

The mean age in group A was 38.6 ± 15.85 years, while in group B was 34.63 ± 14.87 years, with an overall mean of 36.61 ± 15.37 years. This is comparable to the study done by Kumar et al., the mean age in group A (SL) and group B (DL) was 38.69 years and 34.35 years, respectively [5].

Males predominated our study population. Forty-three patients (71.7%) of the total 60 were males. This was, however, found to be statistically insignificant. This finding is in accordance with the preponderance of males in the study sample conducted by Patil et al., in which 70% of the patients were male [6]. All of the studies mentioned above have a predominance of male patients in both the groups compared (and by extension in the total study sample).

In a study by Saboo et al., the mean age for the SL group was 61.53 ± 12.19 kg, and for the DL group was 61.66 ± 9.26 kg. In the same study, the mean hemoglobin for patients in the SL group was 11.25 ± 1.36 g/dL and in the DL group was 11.49 ± 1.24 g/dL, the mean TSP for patients in the SL group was 6.27 ± 0.60 g/dL and in the DL group was 6.53 ± 0.58 g/dL, and the mean S.Alb for patients in the SL group was 3.25 ± 0.21 g/dL and in the DL group was 3.27 ± 0.18 g/dL. In this study, the difference between the means of the two groups was not significant for weight, Hb, TSP, and S. Alb [7]; however, in our study, the difference in the means between the two groups was found to be statistically significant (p=0.002).

The most common indication for GIA was status ileostomy in both groups, with 12 patients each in both groups (40% of the total study population). It was followed by blunt trauma abdomen in 10 patients (17%), strangulated hernia in six patients (10%), perforation peritonitis as a complication of enteric fever in five patients (8%), and Ca descending colon in three patients (5%). Ca caecum, intussusception, Meckel’s diverticulitis, Ca ascending colon, Ca rectum, Ca transverse colon, jejunal stricture, mesenteric infarct, and perforation peritonitis as a result of parasitic infections were rarer diagnoses in the patients in our series. The cases diagnosed (on CECT abdomen) with abdominal/ileocecal TB were managed non-operatively (with conservative measures) with ATT, or an ileostomy was fashioned to relieve the intestinal obstruction. In a study by Kumar et al., maximum numbers of participants in group SL were diagnosed with traumatic intestinal perforation (30.9%). Also, most of the DL group participants were diagnosed with traumatic intestinal perforation (34.6%) [5]. The second most common diagnosis in group SL was sigmoid volvulus (19.2%) and intestinal perforation due to enteric fever (19.2%), while in group DL, the second most common diagnosis was sigmoid volvulus (26.9%) [5]. The most common condition requiring GIA in the study by Sharma et al. was a terminal ileal perforation in a total of 12 out of 50 patients (24%). The next most common indication was status ileostomy in 10 out of the 50 patients (20%) [8]. In a study of 50 cases by Patro et al. (n=50), resection of the terminal ileum and ileoileal anastomosis was performed in a maximum number (26%) of patients [9]. The next most common condition encountered in the study was ileocecal tuberculosis (14%). Malignant conditions, taken together, made up 30% of the cases [9].

The most common type of anastomosis done in our study was an entero-enteric (EE) type and the least commonly done was a colo-colic (CC) anastomosis. This is in agreement with the studies by done Patil et al., Kumar et al., Sharma et al., and Patro et al., where EE anastomoses were the most commonly done anastomosis [5,6,8,9]. In all the studies mentioned above, only the study conducted by Kumar et al. reported two patients undergoing a colorectal (CR) anastomosis for Ca rectum as a part of a low anterior resection (LAR) [5].

The mean (±SD) duration of anastomosis for the SL group was 19.57 ± 2.25 minutes, for the DL group was 30 ± 2.59 minutes, the difference in the duration of anastomosis was statistically significant (p=0.002). This is comparable to the study done by Kumar et al. where the mean duration for the SL group was 19.54 ± 1.63 minutes and for the DL group was 28.12 ± 1.68 minutes (p<0.005) [5]. This study also fairly tallies with the studies by Patil et al., Owaid et al., Patro et al., Saboo et al., Sharma et al., and Dhamnaskar et al., all reporting a statistically significantly less time to fashion an SL as compared to a DL anastomosis [7-11].

A meta-analysis of 6 RCTs done by Shikata et al. in 2006 reported arithmetic mean duration of 23.4 minutes for SL and 36.9 minutes for DL anastomosis (for the study by Burch et al. in 2000) [12,13]. They commented that considering the reduced duration (and medical expenses) to fashion an SL anastomosis, it may prove to be the optimal choice in most surgical situations [12,13]. In a systematic review of a trial (Burch 2000) by Sajid et al., they concluded that the average operative time for SL GIA was significantly shorter than the DL GIA [14].

The mean duration of hospital stay in the SL group was 8.97 ± 3.08 days and in the DL group was 8.93 ± 2.61 days, the difference was found to be statistically insignificant. Of the four patients with AL in our study, the three patients that recovered following conservative/operative intervention had, as expected, a protracted hospital stay (18-21 days). This is in line with the study done by Kumar et al. who reported no statistically significant difference in the duration of hospital stay between the two groups, although the duration was slightly higher in the DL group (11.58 ± 1.528 minutes) than SL group (11.08 ± 1.055 minutes) [5].

However, in the study by Sharma et al. (SL 5.74 days and DL 7.70 days [p=0.000]), Owaid et al. (SL 7.00 ± 1.778 days and DL 9.74 ± 1.990 days [p=<0.001]) and Dhamnaskar et al. (SL 8.84 ± 3.11 days and DL 10.44 ± 5.87 days [p=0.048]), the difference in the mean duration of hospital stay between the two groups was found to be significant [8,10,11].

The most common complication in our study was wound infection, seen in a total of 11 (18%) patients (four patients in the SL and seven in the DL group), followed by wound dehiscence in six (10%) patients (three patients in each group). Mittal et al. in their study of 60 cases of ileostomy reversal reported a 20% wound infection (most common complication overall) and an 8.3% wound dehiscence rate [15]. The combined incidence of post-operative abscesses and fistulae in our study was 10% (six patients in total, four in the SL, and two in the DL group). Mittal et al. reported an 8.3% occurrence of post-operative intra-abdominal collections [15]. Sharma et al. reported a single patient of post-op intra-abdominal abscess in their study [8].

In our study, three patients from the DL group developed postoperative incomplete intestinal obstruction. This was differentiated from a prolonged postoperative paralytic ileus by a return of bowel sounds and passage of flatus on from post-operative day 2 (POD-2) and seen clinically as abdominal distention, dull abdominal pain, and nausea (despite adequate intravenous anti-emetic administration) on and from POD-3. Electrolyte abnormalities, uremia, and sepsis were ruled out on the basis of normal blood biochemistry and the absence of clinical signs and symptoms. X-ray whole abdomen erect and supine views were done along with USG whole abdomen. Radiological investigations revealed dilated gut loops with USG suggestive of constriction at the site of anastomosis. Patients were managed conservatively with intravenous anti-emetics and analgesics. Ryle’s tube was inserted and active aspiration was done every four hours. All the patients responded well to conservative management. This peculiar finding in our study can be postulated to be a result of the difficulty in teaching the DLGIA technique to surgical residents as compared to SLGIA. In other DLGIAs performed by more experienced surgeons, and in fact, in SLGIAs fashioned by surgical residents, no such complication was seen. In contrast. a study by Burch et al. remarked that the SLGIA technique can be incorporated into a surgical training program without a significant increase in complications [13].

The overall incidence of AL in our study was 7% (two patients in each SL and DL group). All the patients in our study that developed AL had a pre-operative S. Alb concentration below 3.4 g/dL (range: 2 g/dL-2 .38 g/dL). In a systematic review of RCTs (Burch 2000) by Sajid et al., they concluded that the risk of anastomotic dehiscence following SL GIA was slightly less than DL GIA, but statistically, it was not significant [14]. On further sensitivity analysis of three of the above-mentioned high-quality trial (Burch 2000), they found that the risk of anastomotic dehiscence following SL GIA was as high as in DL GIA [14].

In our study, one patient with AL in the DL group expired following (mortality of 1.6%) while the three other patients with AL were re-explored and had an otherwise uneventful hospital stay and an unremarkable follow-up. In the study by Patro et al., mortality of 2% was reported for the patient who had developed an AL (two patients from the DL group had an AL, one recovered and one expired) [9]. While Nemma et al. reported two mortalities, both from the DL group, Saboo et al. reported higher mortality in the DL group (2/30 patients) than SL group (1/30 patients), both reporting a total of 5% mortality [7,16]. In a systematic review by Sajid et al., reporting the mortality data, they concluded that the risk of mortality following SL GIA and DL GIA was statistically the same [14].

## Conclusions

Thus, in our study, the single-layer method of intestinal anastomosis resulted in a significant reduction in time; without any difference in complications. It also is comparatively easier to train surgical residents in the single-layer technique employed in our study than the double-layered approach and this is particularly important in the setting of a teaching institute. The present study advocates the use of the single-layer method for intestinal anastomosis.
